# Intellectual disability and autism prevalence in Western Australia: impact of the NDIS

**DOI:** 10.3389/fpsyt.2024.1359505

**Published:** 2024-05-20

**Authors:** Jenny Bourke, Richard Sanders, Jocelyn Jones, Maathumai Ranjan, Kingsley Wong, Helen Leonard

**Affiliations:** ^1^ Child Disability, Telethon Kids Institute, Nedlands, WA, Australia; ^2^ Sanders Consulting WA, Education Consultancy, Perth, WA, Australia; ^3^ Crawford School of Public Policy, Australian National University, Canberra, ACT, Australia

**Keywords:** intellectual disability, autism, developmental disorder, prevalence, trends

## Abstract

**Introduction:**

Estimates of the prevalence of intellectual disability or autism spectrum disorder (ASD) may vary depending on the methodology, geographical location, and sources of ascertainment. The National Disability Insurance Scheme (NDIS) in Australia was introduced progressively from 2016 to provide individualized funding for eligible people with a significant and permanent disability.

**Methods:**

Its recent inclusion as a source of ascertainment in the population-based Intellectual Disability Exploring Answers (IDEA) database in Western Australia has allowed comparisons of the prevalence of intellectual disability and ASD before and after its introduction.

**Results:**

Prevalence of intellectual disability in 2020 was 22.5 per 1,000 (/1,000) live births compared with previous estimates in 2010 of 17/1,000, and for ASD, the estimate was 20.7/1,000 in 2020 compared with 5.1 /1,000 in 2010. Whilst the prevalence of ASD in Aboriginal individuals was about two-thirds that of non-Aboriginals, there was an increased prevalence of ASD in Aboriginal children under 10 years compared with non-Aboriginal children.

**Discussion:**

The concurrent relaxation of ASD diagnostic practice standards in Western Australia associated with the administration of access to the NDIS and the release of the National Guidelines empowering single diagnosticians to determine the appropriateness of engaging additional diagnosticians to form a multidisciplinary team on ASD diagnosis, appear to be important factors associated with the increase in ASD diagnoses both with and without intellectual disability.

## Introduction

Intellectual disability is a neurodevelopmental disorder characterized by impaired intelligence that results in significant deficits in adaptive functioning, manifesting before the age of 18 years (1–3). The clinical diagnosis of intellectual disability typically employs individually administered, standardized measures of intelligence appropriate for an individual’s culture and language, the results of which are interpreted with clinical judgement, sensitive to the limitations of the psychometric properties of the measures and corroborated with qualitative information across contexts to be considered valid. Intellectual impairment is defined relative to the population estimates (or more typically references a normative sample of the measures used) and appropriately classified where estimates of intelligence are approximately 2 standard deviations or more below the mean. A similar level of impairment in adaptive functioning (or subcomponents) and an onset prior to 18 years could see intellectual disability considered an appropriate clinical diagnosis for an individual (1, 2). Historically, the severity of an intellectual disability was categorized on the basis of the magnitude of the intellectual impairment: mild intellectual disability for greater than 2 standard deviations, moderate between 3 and 4, and severe when the impairment is greater than 4 standard deviations below the mean. Other than recent iterations of the Diagnostic and Statistical Manual of Mental Disorders (DSM) changing the subcategorization of intellectual disability on the basis of the severity of the resulting adaptive behavior deficits, the criteria for diagnosing intellectual disability have remained remarkably stable over the last 50 years (1). There are many factors that may influence the estimation of the prevalence of intellectual disability in a population. These include the age group and the source of cases from population-based administrative datasets or selective hospital or community sampling. One international meta-analysis of the prevalence of intellectual disability found that the overall prevalence was 10.4 per 1,000 population (4). However, there was considerable variability depending on the study design, with a higher pooled prevalence of 18.3/1,000 in children and adolescents (4).

**Figure 1:**
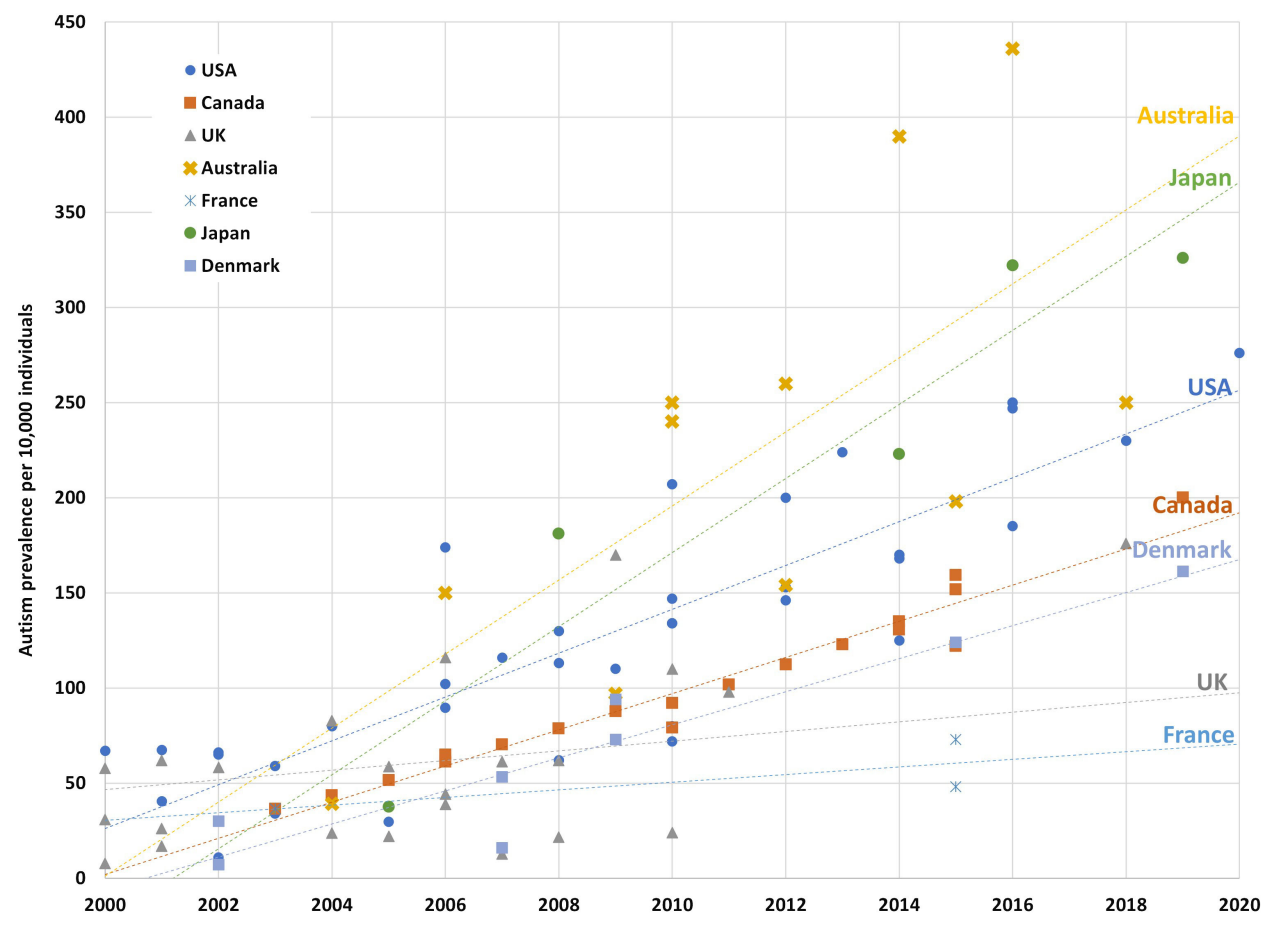
Autism prevalence in studies of children from 2000 to 2020 by country. The figure shows prevalence estimates from longitudinal and cohort studies over the last two decades with the trend line reflecting the line of best fit for each country. The Australian estimates above the trend line are from longitudinal studies using the LSAC database, whereas the estimates below the trendline are from national survey data, which peak bodies believe underestimate true prevalence. Reproduced from Ranjan M. Understanding Autism Prevalence (November 20, 2023). TTPI - Working Paper 17/2023 November 2023. Canberra, ACT: TTPI (2023). doi: 10.2139/ssrn.4638072 (20). Copyright Maathumai Ranjan.

The recognition of autism has developed from initial observances by Kanner of atypical social behaviors in children (5) to now being a “set of heterogeneous neurodevelopmental conditions, characterized by early-onset difficulties in social communication and unusually restricted, repetitive behavior and interests” (6), in general terms. Operational definitions of autism have evolved from DSM-III, based on Rutter’s conceptualization of autism as a developmental disorder rather than a form of childhood psychosis (7). Subsequent revisions in DSM-IV removed the requirement of onset before 30 months and introduced a broader definition of pervasive developmental disorder that emphasized the early onset of a triad of features (8). Further revisions in DSM-5 adopted the umbrella term autism spectrum disorder (ASD) without a definition of subtypes and reorganized the triad into a dyad: difficulties in social communication and social interaction; and restricted and repetitive behavior, interests, or activities (2). Furthermore, atypical language development, which was historically linked to an autism diagnosis, was removed from the criteria and is now classified as a co-occurring condition. In Australia, a diagnosis of ASD is determined using the DSM diagnostic criteria (9, 10) but due to observed variation in diagnostic practices, including the choice of accompanying assessment tools (11), recommendations for national standards have now been developed (10). One recent Australian study using data linkage between three routinely collected datasets (disability services, hospital admissions, and mental health records) estimated ASD prevalence by age 12 years at 1.3% (12). The prevalence of ASD worldwide has increased over time with considerable variability depending on methodology, geographical location, and sources of ascertainment (13–16). Whilst a global meta-analysis estimated 0.4% in Asia, 1% in America, 0.5% in Europe, and 1.7% in Australia (17), a systematic review using published data from various regions found a median prevalence of 10/1,000 (1%) (18) while longstanding studies such as the Centers for Disease Control and Prevention in the US currently estimate 1.7% in four-year-olds (19). Despite the variability of estimates being impacted by the differences in data collection methods, data sources, and data quality, the prevalence for ASD is generally accepted to be at least 1% using administrative databases and higher when population screening is employed. A recent study comparing global trends in prevalence noted that estimates of prevalence in Australia are some of the highest in the world (**Figure 1**) (20). Further, the growth in estimates of autism prevalence in children over the past two decades is steeper in Australia than in other countries with comparable economic and health profiles.

In Australia, the National Disability Insurance Scheme (NDIS), administered by the National Disability Insurance Agency (NDIA), was introduced to provide individualized funding for eligible people with a significant and permanent disability to improve their access to support services and to gain greater independence and improved social, educational and economic participation (21). The scheme was trialed across specific locations in Australia from July 2013 and then progressively introduced nationally from July 2016. An independent trial was conducted in Western Australia (WA) in July 2014, where statewide services had previously been provided through the WA Disability Services Commission (DSC). The transition from DSC to the nationally-administered NDIS occurred from December 2017 to June 2023 (22). In contrast to the relative stability of diagnostic practice in intellectual disability, administrative transfer from the DSC to the NDIA in WA may have had the unintended consequence of broadening the diagnostic practices for ASD, enabling more individuals to access disability support. Historically, WA has been recognized as having consistent and high standards in the diagnosis of ASD (11). In the 1990’s DSC aligned with the Western Australian Autism Diagnosticians’ Forum’s guidelines which required, amongst other hallmarks of good practice, multidisciplinary teams in a central panel of assessors to be involved in the diagnosis of ASD (9). Further, eligibility for State-funded disability services was previously determined by clinical staff with extensive expertise in the diagnosis of ASD. Transition to the NDIA access criteria allowed for diagnoses given by single diagnosticians such as pediatricians, who are not required to but often consult with a multidisciplinary team, to secure access to disability supports. Whilst the NDIA employs access assessors to review evidence of disability, they generally do not hold specialist expertise in the diagnosis of ASD commensurate with their counterparts in the outgoing State administration. Further destabilization of diagnostic practice in WA resulted from the release of the National Guidelines for assessment and diagnosis of autism spectrum disorders (10). The guidelines legitimized a wider range of diagnostic assessment practices as appropriate and empowered single diagnosticians to determine the appropriateness of engaging additional diagnosticians to form a multidisciplinary team.

Using DSC and Department of Education WA data, we previously found that the prevalence of intellectual disability among WA Aboriginal and Torres Strait Islander people was more than twice that for non-Aboriginal people, and children who did not engage with formal disability services were more than five times likely to be Aboriginal (23), suggesting disparities in both diagnostic pathways and engagement with support services. The concept of disability for Aboriginal people is complex and often culturally at odds with the Western biomedical model of disability (24). The barriers to accessing disability services may be associated with issues related to cultural accessibility and the availability of services relevant to their needs (25). The recent report on First Nations people with disability from the Royal Commission into Violence, Abuse, Neglect and Exploitation of People with Disability noted on the Aboriginal perspective of disability, “there is a stark difference in how our people view what is now commonly referred to as disability. First Nations peoples have different values, beliefs and social practices about health, wellbeing and how the body functions” (26). Additionally, it was noted a reluctance to seek diagnosis due to associated labeling often results in Aboriginal people in Australia being unaware of the services and supports available to people with disability, including through the NDIS. Alternatively, it may reflect a wariness of overdiagnosis of neurodevelopmental disorders expressed by some Aboriginal people (27).

The potential impact of the introduction of the NDIS on the estimated population prevalence of intellectual disability and ASD in Australia can be demonstrated using data from the WA population-based Intellectual Disability Exploring Answers (IDEA) database (23). The source of ascertainment may be related to the level of impairment, with more severely affected children more likely to be ascertained through disability services, and milder children through the Department of Education (23). IDEA is a population-based collection of people diagnosed with intellectual disability, ASD, or both in WA with ascertainment of cases linked from statewide service and diagnostic data (DSC and Department of Education WA) and more recently available national service data (i.e., NDIS). The aim of this study was to investigate differences in WA prevalence estimates of intellectual disability and ASD before and after the addition of NDIS data, to understand changes in diagnostic and service-driven trends at the population level.

## Materials and methods

### Study population

Live births in WA between 1 January 1983 and 31 December 2015 formed the cohort for the study and thus individuals were aged 5-37 years at the time of data extraction. Since 1999, using data linkage among datasets from the Midwives Notification System (MNS), Disability Services Commission (DSC), and Department of Education WA (Education), individuals with a diagnosis of either intellectual disability or ASD were identified as eligible for the Intellectual Disability Exploring Answers (IDEA) database (28). The additional linkage of all WA cases identified with intellectual disability or ASD through the National Disability Insurance Agency (NDIA) occurred in 2020. Notifications from private and Catholic schools were linked in 1999 for birth years 1983-1992 (29), and so some individuals born after 1992 who were ascertained by these independent schools may not be included in IDEA if they did not access either DSC or NDIS services.

Cases ascertained through DSC, the organization that provided services to the intellectual disability population from 1953 until the introduction of the NDIS, were included in IDEA if they had a full-scale IQ of less than 70 and met eligibility for intellectual disability (30); they had a condition known to be consistent with intellectual disability (such as Down syndrome) or a review of medical records had documented them as having intellectual disability. The severity level of intellectual disability for IDEA cases is defined as mild (IQ 55–69), moderate (IQ 40–54), or severe (<40). Cases identified only through Education were considered eligible if they had a level of intellectual disability defined as either mild/moderate or severe. However, from 2006, the information provided on the severity of intellectual disability for these cases was replaced with a category of educational need, coded from 1 (low need) to 5 (high need), later replaced from 2016 onwards with an Individual Disability Allocation scaled from 1 (low impact of disability) to 7 (severe impact) (23).

NDIA cases were eligible if they had been assigned an International Classification of Diseases (ICD-10-CM) diagnostic code associated with intellectual disability (F70-F73, F79) or a condition known to be consistent with intellectual disability. The severity level of intellectual disability for NDIA cases was coded as mild, moderate, or severe in accordance with the ICD code or the NDIA severity score (1-5 mild, 6-10 moderate, and 11-15 severe).

Information on ASD diagnosis was available through DSC or NDIA. In Education data, there is a code to indicate the main disability as either being intellectual disability or ASD, and this was also used to determine an ASD diagnosis. ASD cases with a mild or moderate, severe or unknown but confirmed level of intellectual disability were coded as having ASD with intellectual disability.

The maternal Aboriginal and Torres Strait Islander (Indigenous) status flag, which uses a validated algorithm based on a person’s Aboriginal status in a number of administrative datasets (31), was provided to IDEA during the data linkage process. Metropolitan and country health regions can be designated through postcodes. Based on available postcode information through DSC or NDIA, or school location if the data was not available, individuals were grouped into one of 8 health regions: 7 of which are under the WA Country Health Service (Kimberley, Pilbara, Midwest, Goldfields, Wheatbelt, South West, and Great Southern) and metropolitan areas were combined into one region.

### Statistical analysis

Descriptive statistics were used to summarize the characteristics of the birth cohort. The prevalence of intellectual disability was defined as the proportion of all live births in WA from 1983 to 2015 who were diagnosed by 2020. Limiting the birth years from 1983 to 2015 allows for a minimum of 5 years of follow-up, enabling ascertainment through Education sources. The establishment in WA in 1991 of a multidisciplinary Central Diagnostic Panel to review the diagnostic assessment of ASD, coincided with a marked increase in the annual incidence of ASD (32). Therefore, the prevalence estimates for ASD were limited to number of cases for birth years 1993 to 2015, when the diagnostic processes for ASD became more regulated. All prevalence values were expressed per 1,000 live births, and their 95% exact binomial confidence intervals were derived using the Clopper-Pearson estimation method (33). The prevalence ratios and their 95% confidence intervals were determined using the Stata ‘‘csi’’ command, in which the standard error was estimated using the method by Greenland and Rothman (34).

The incidence of intellectual disability or ASD was calculated based on the number of cases diagnosed every two years from 1983 to 2020 for intellectual disability or from 1993 to 2020 for ASD, divided by the estimated birth cohort in WA at risk during the relevant period. The result was expressed per 10,000 person-years at risk. The numerator was the number of newly diagnosed cases of intellectual disability or ASD in each period, using a proxy year of diagnosis based on the year of entry to DSC, the year of ascertainment from the Department of Education, or the year of entry to NDIS for those newly ascertained through NDIA. The denominator was all individuals in the birth cohorts at risk of diagnosis since birth, assuming no loss to death or migration. Birth cohort estimates were obtained from the Australian Bureau of Statistics (ABS) (35).

All analyses were performed using Stata version 17.0 (StatCorp, College Station, TX, USA).

## Results

There were 890,937 individuals born in WA between 1 January 1983 and 31 December 2015, ranging from approximately 22,875 per birth year in 1983 to 34,757 per birth year in 2015. Of these, 20,046 individuals were identified with an intellectual disability through the IDEA database by 2020: 68.2% were male, and 31.8% were female. An additional 5,441 individuals were identified with ASD without intellectual disability.

**Figure 2:**
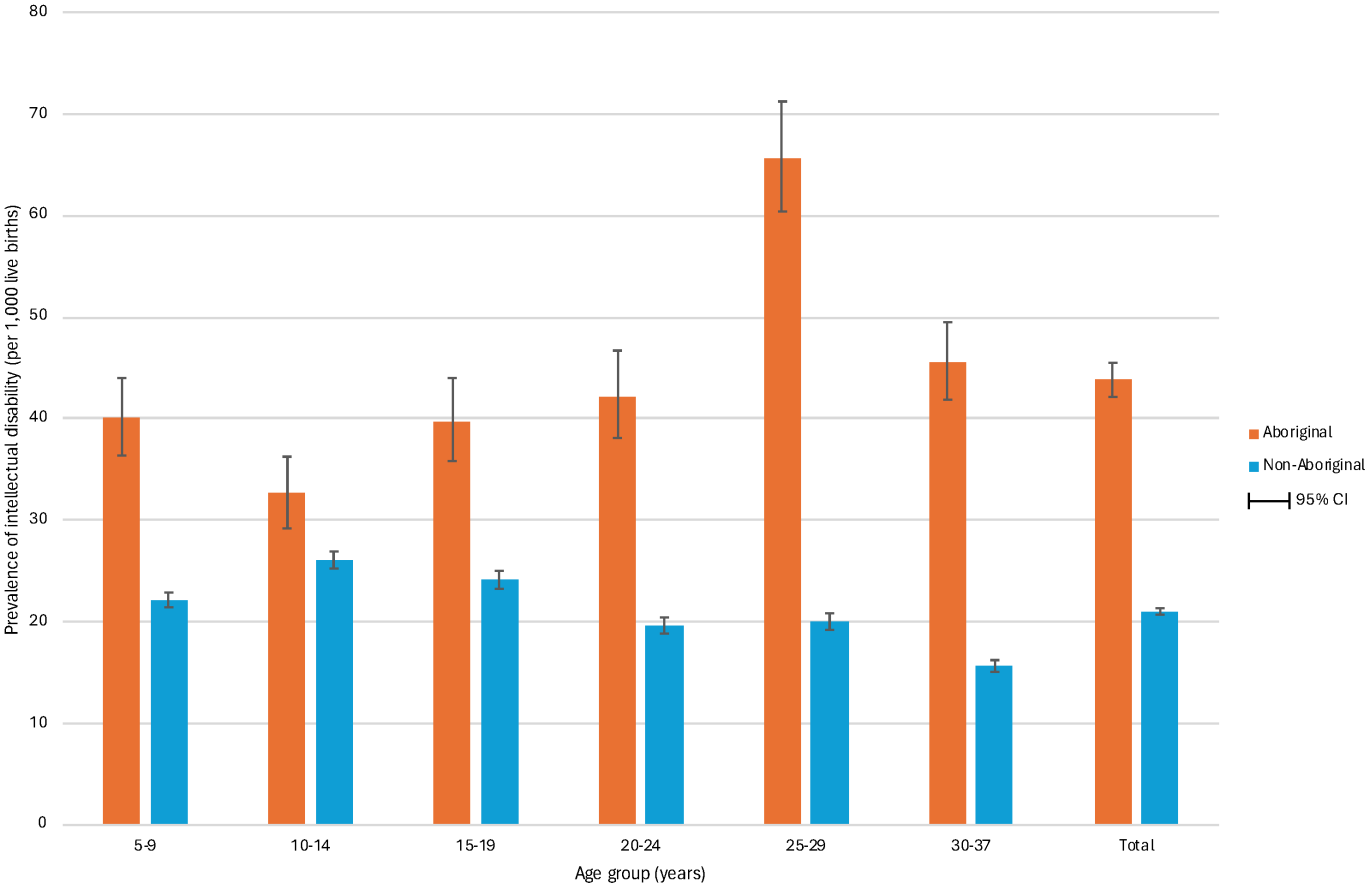
Prevalence of intellectual disability by age and maternal indigenous status in 890,837 individuals born in Western Australia between 1983 and 2015 and followed to 2020. The figure shows the prevalence of intellectual disability per 1,000 live births by age group for Aboriginal and non-Aboriginal individuals. The prevalence was highest in the 25-29 years group for Aboriginal individuals (65.7, 95% CI 60.4,71.2) and in the 10-14 years group for non-Aboriginal individuals (26.1, 95% CI 25.2,26.9). Moreover, the prevalence was higher in all age groups for Aboriginal individuals compared to non-Aboriginal individuals.

The prevalence of intellectual disability was estimated to be 22.50 per 1,000 live births, with the mild or moderate and the severe subgroups at 21.06/1,000 and 1.44/1,000, respectively (**Table 1**). This compares with previous published WA estimates followed up to 2010 of 17/1,000 (23). Prevalence varied considerably, with males more than twice as likely to have intellectual disability (Prevalence Ratio [PR] 2.1, 95% CI 2.04, 2.17) compared with females. Additionally, Aboriginal individuals had a prevalence of 43.78/1,000 compared with 21.01/1,000 in the non-Aboriginal cohort (PR 2.08, 95%CI 2.00, 2.17). The prevalence of intellectual disability also varied by age, with the highest prevalence in Aboriginal individuals aged 25-29 years (**Figure 2**).

**Table 1:** Prevalence of Intellectual disability (per 1,000 live births) in 890,837 individuals born between 1983 and 2015 who were followed up to 2020, by maternal Indigenous status, sex and severity.

	Population	Mild or Moderate			Severe			Total		
		n	Prevalence (95% CI)	PR (95%Cl)	n	Prevalence (95% CI)	PR (95%CI)	n	Prevalence (95% CI)	PR (95%CI)
Overall										
Male	456,908	12,896	28.23 (27.75.28.71)	2.07* (2.01,2.14)	782	1.71 (1.59,1.84)	1.49* (1.34,1.67)	13,680	29.94 (29.45,30.44)	2.10* (2.04,2.17)
Female	434,029	5,823	13.41 (13.07.13.76)	Ref	497	1.15 (1.05,1.25)	Ref	6,320	14.56 (14.21.14.92)	Ref
Total	890,937	18.764^a^	21.06 (20.76.21.36)	N/A	1,282^b^	1.44 (1.36,1.52)	N/A	20.046	22.50 (22.19,22.81)	N/A
Aboriginal										
Male	29,639	1,595	53.81 (51.27.56.44)	2.03^ (1.93.2.14)	116	3.91 (3.24.4.69)	2.51^ (2.06.3.06)	1,711	57.73 (55.10.60.44)	2.06^ (1.96,2.16)
Female	28,495	761	26.71 (24.86.28.85)	2.14^ (1.98,2.31)	67	2.35 (1.82.2.98)	2.22^ (1.72.2.87)	828	29.06 (27.14.31.07)	2.15^ (2.00, 231)
Total	58,134	2,361^d^	40.61 (39.02.42.25)	2.06^ (1.98,2.15)	184^e^	3.17 (2.72,3.66)	2.40^ (2.06.2.81)	2,545^f^	43.78 (42.13.45.47)	2.08^ (2.00, 2.17)
Non-Aboriginal										
Male	427,269	11,303	26.45 (25.08.26.94)	Ref	666	1.56 (1.44,1.68)	Ref	11,969	28.01 (27.52,28.51)	Ref
Female	405,534	5,062	12.48 (12.14.12.83)	Ref	430	1.06 (0.96.1.16)	Ref	5,492	13.54 (13.19,13.90)	Ref
Total	832,803	16,403	19.69 (19.40.20.00)	Ref	1,098^h^	1.32 (1.24.1.40)	Ref	17,501^i^	21.01 (20.71.21.32)	Ref

n, number of cases; CI, confidence interval; PR, prevalence ratio; Ref, reference category; N/A, not applicable.

*comparing male to female; ^comparing Aboriginal to non-Aboriginal.

number of cases with missing sex variable data: ^a^n=43; ^b^n=3; ^c^n=46; ^d^n=5; ^e^n=1; ^f^n=6; ^g^n=38; ^h^n=2; ^I^n=40.

**Figure 3:**
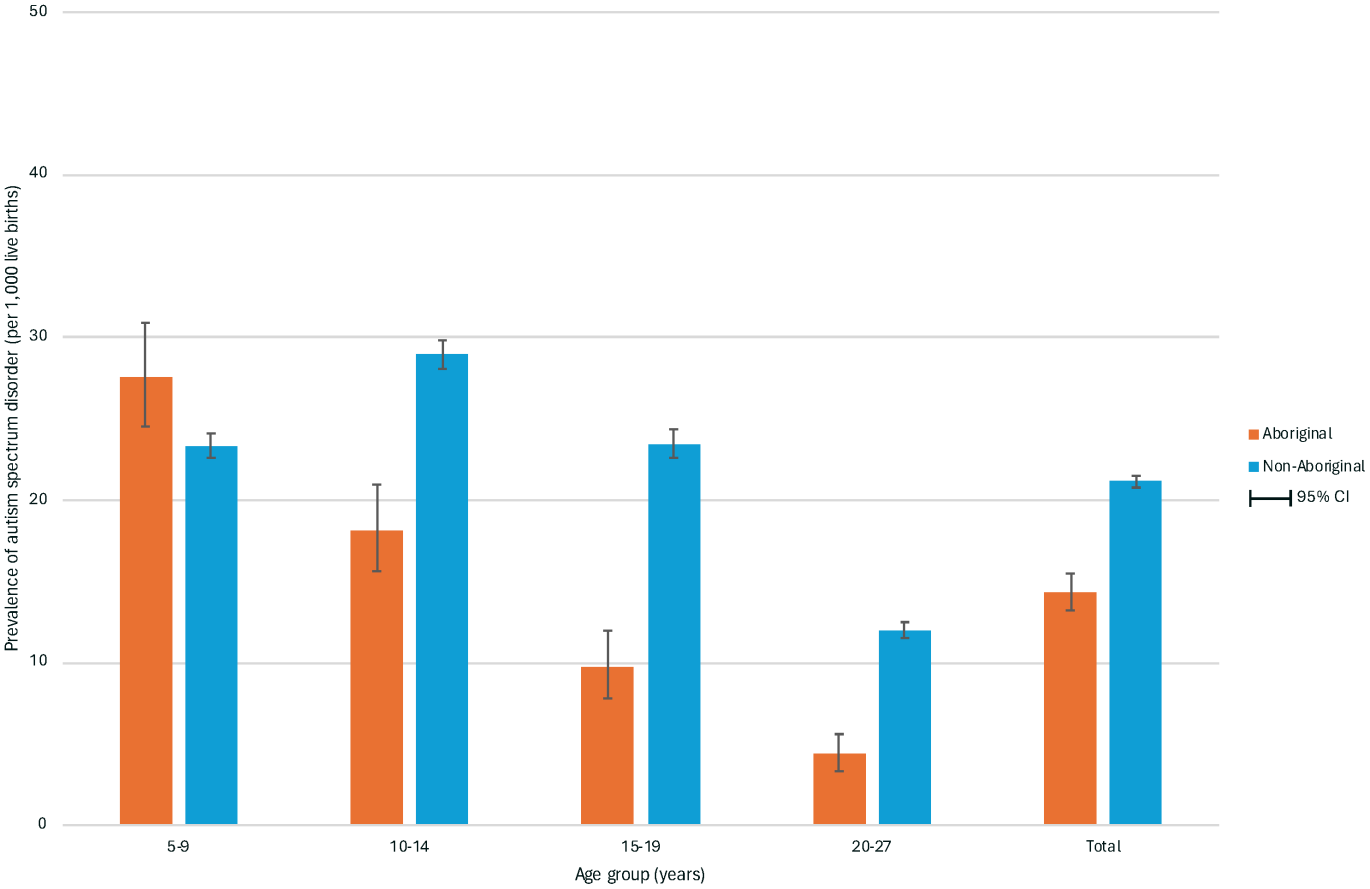
Prevalence of autism spectrum disorder by age and maternal indigenous status in 648,247 individuals born in Western Australia between 1993 and 2015 and followed to 2020. The figure shows the prevalence of autism spectrum disorder per 1,000 live births by age group for Aboriginal and non-Aboriginal individuals. The prevalence was highest in the 5-9 years group for Aboriginal individuals (27.6, 95% CI 24.5,30.9) and in the 10-14 years group for non-Aboriginal individuals (28.9, 95% CI 28.1,29.8). Apart from the youngest age group, the prevalence was higher in all age groups for non-Aboriginal individuals compared to Aboriginal individuals.

**Figure 4:**
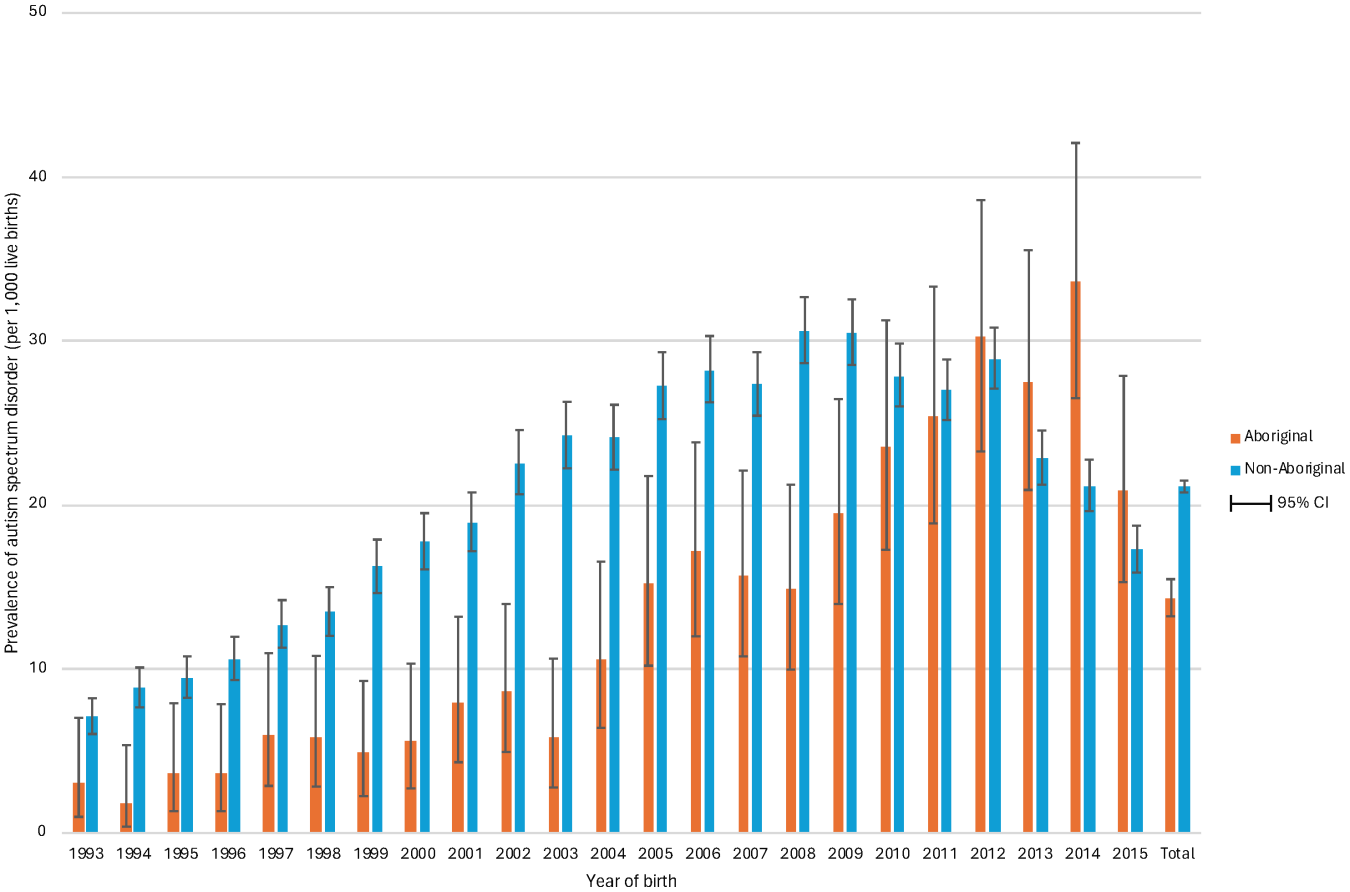
Prevalence of autism spectrum disorder by birth year and maternal indigenous status in 648,247 individuals born in Western Australia between 1993 and 2015 and followed to 2020. The figure shows the birth year trends in the prevalence of autism spectrum disorder (ASD) among Aboriginal and non-Aboriginal individuals. The prevalence of ASD, expressed per 1,000 live births, increased over birth years and peaked above 30 among non-Aboriginal individuals born in 2008 and 2009 and in Aboriginal individuals born in 2012 and 2014. The prevalence was consistently higher in non-Aboriginal populations compared to Aboriginal individuals until the 2010s, when there was a reversal, most notably among those born in 2014.

Of individuals born between 1983 and 2015, 14,332 were diagnosed with ASD: 8,891 (62.0%) with comorbid intellectual disability (ASD with ID) and 5,441 (38.0%) without intellectual disability (ASD without ID). When limiting the birth years to between 1993 and 2015, when ASD diagnoses became more regulated, the proportions were very similar (62.5% and 37.5%, respectively). Over this period, the prevalence of ASD was estimated to be 20.67/1,000; 30.89/1,000 in males and 9.71/1000 in females (PR 3.18, 95%CI 3.06, 3.31) (**Table 2**). This indicates that the prevalence of ASD has increased considerably since previous estimates up to 2010, which showed 5.1/1,000 for ASD, and among them, 3.8/1,000 with intellectual disability, and 1.3/1,000 without intellectual disability (23). By indigenous status, the prevalence of ASD was 14.31/1,000 in Aboriginal individuals and 21.12/1,000 in the non-Aboriginal cohort (PR 0.69, 95%CI 0.64, 0.74). Whilst the prevalence of ASD was higher in non-Aboriginal individuals, the prevalence was greater in young Aboriginal people under 10 years of age (**Figures 3**, **4**).

**Table 2:** Prevalence of autism spectrum disorders (per 1,000 live births) in 648,247 individuals born between 1993 and 2015 who were followed up to 2020, by maternal Indigenous status and sex.

	Population	ASD			Prevalence (95% CI)	PR (95% CI)
		With ID, n	Without ID, n	Total, n		
Overall						
Males	331,999	6,498	3,759	10,257	30.89 (30.31, 31.49)	3.18* (3.06, 3.31)
Females	316,248	1,843	1,228	3,071	9.71 (9.37, 10.05)	Ref
Total	648,247	8,376^a^	5,024^b^	13,400^c^	20.67 (20.33, 21.02)	N/A
Aboriginal						
Males	22,065	377	116	493	22.34 (20.43, 24.38)	0.72^ (0.66, 0.78)
Females	21,048	85	34	119	5.65 (4.69, 6.76)	0.58^ (0.49, 0.69)
Total	43,113	466^d^	151^e^	617^f^	14.31 (13.21, 15.48)	0.69^ (0.64, 0.74)
Non-Aboriginal						
Males	309,954	6,121	3,643	9,764	31.50 (30.89, 32.12)	Ref
Females	295,180	1,758	1,194	2,952	10.00 (9.644, 10.37)	Ref
Total	605,134	7,910^g^	4,873^h^	12,783^i^	21.12 (20.76, 21.49)	Ref

ASD, autism spectrum disorder; ID, intellectual disability; n, number of cases; CI, confidence interval; PR, prevalence ratio; Ref, reference category; N/A, not applicable.

*comparing male to female; ^comparing Aboriginal to non-Aboriginal.

number of cases with missing sex variable data: ^a^n=35; ^b^n=37; ^c^n=72; ^d^n=4; ^e^n=1; ^f^n=5; ^g^n=31; ^h^n=36; ^I^n=67.

**Figure 5:**
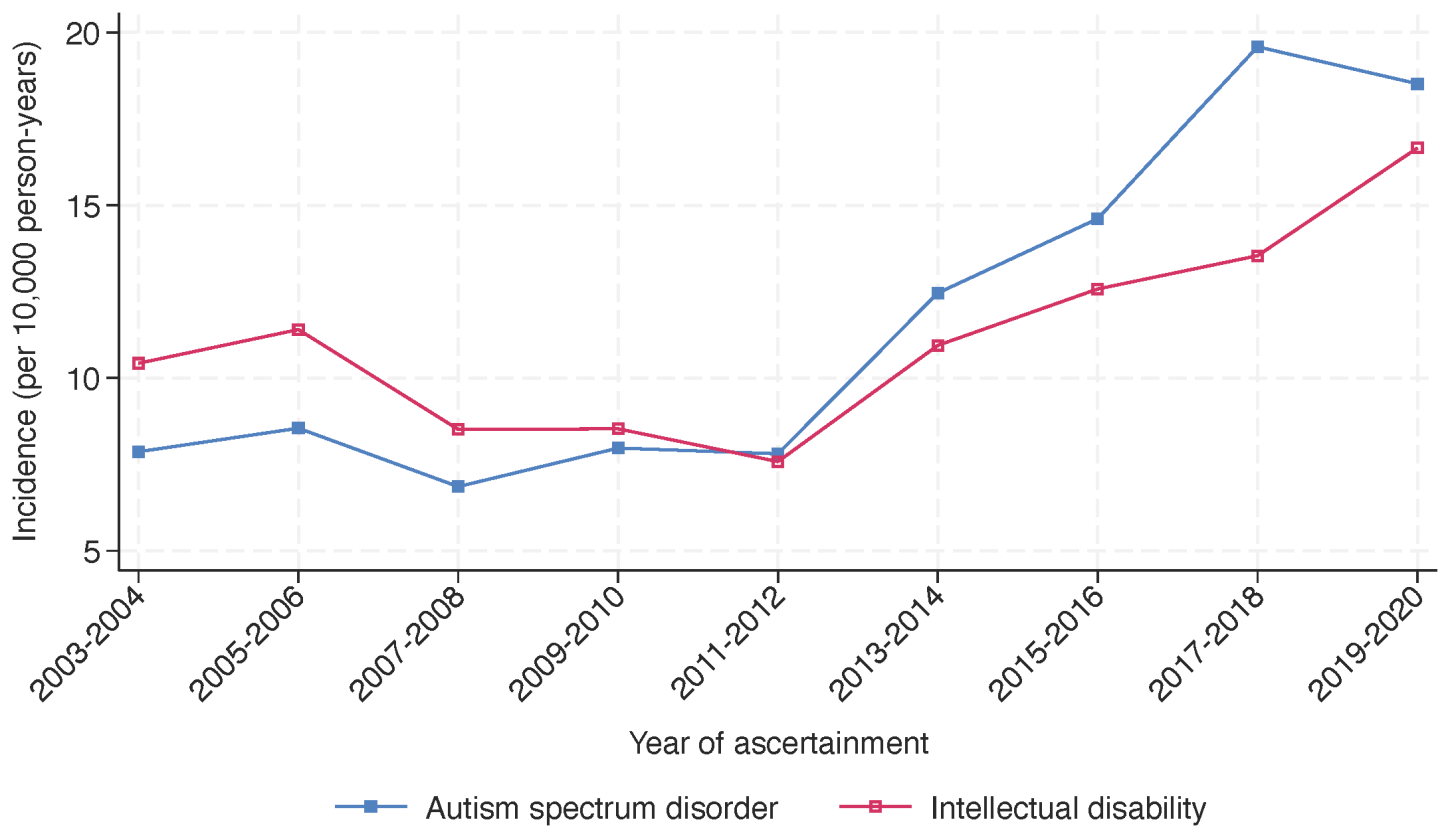
Incidence of autism spectrum disorder and intellectual disability in Western Australia, 2003-2020. The figure illustrates the incidence of autism spectrum disorder (ASD) and intellectual disability in Western Australia from 2003 to 2020. Incidences are presented per 10,000 person-years and were assessed biennially over the study period. While both conditions exhibit a general upward trend, the incidence of ASD shows a steeper increase in the later years (from 2011-2012) compared to intellectual disability.

**Figure 6:**
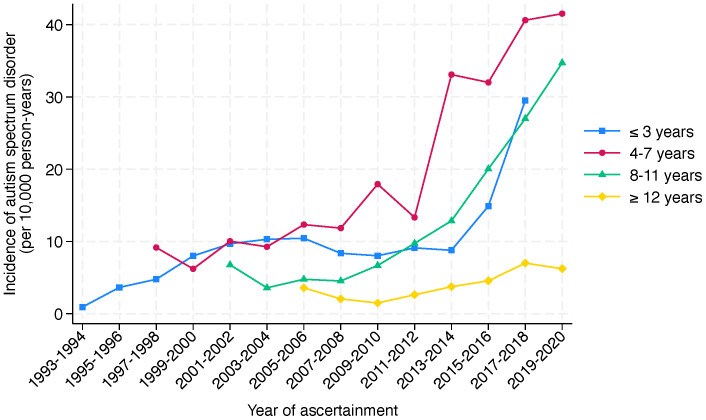
Incidence of autism spectrum disorder by age group in Western Australia, 1993-2020. The figure presents the incidence of autism spectrum disorder per 10,000 person-years in Western Australia, segmented by age groups, over a period of ascertainment spanning from 1993 to 2020. The incidence is divided into four age categories: ≤3 years (light blue), 4-7 years (red), 8-11 years (green), and ≥12 years (yellow). The graph reveals a clear trend of increasing incidence across all age groups over a 27-year period of ascertainment. Notably, the incidence in the 4-7 years age group shows a pronounced rise from 2011-2012. A similar change is observed for the ≤3 years and 8-11 years age groups from 2013-2014.

The incidence of ASD and intellectual disability over the period between 2003 and 2020 (**Figure 5**) shows a steady incidence of ASD from 2003 to 2012, after which the incidence increased more sharply. In contrast, the incidence of intellectual disability declined over the early period before increasing, to a lesser degree than that of ASD, after 2012. The incidence of ASD by age group shows that the incidence was highest in the 4 to 7-year group (**Figure 6**).

### Ascertainment source

Cases in IDEA born 1983-2015 may be ascertained from multiple sources, as shown in **Table 3**. Of the 25,487 individuals, 5,442 (21.4%) had been ascertained only through education sources and 2,104 (8.3%) only through NDIA. The introduction of the NDIS involved individuals previously registered with DSC transitioning to the NDIS. There was a considerable overlap of ascertainment sources, indicating that some received additional services through DSC or NDIA, and some were ascertained through education sources alone. There were 5,899 (23.1%) individuals receiving NDIS services in 2020 but not previously registered with DSC, of whom 3,795 were also identified through Education, about half of whom (n=2,149, 56.7%) had been previously (prior to 2020) ascertained in IDEA through Education sources only. Of the 5,899 individuals, 5,144 (86.7%) had been diagnosed with ASD, and among them, 3,097 with intellectual disability, and 2,047 without. The frequency distribution of these individuals receiving NDIS services in 2020 by age group, indigenous status, health region, and disability subgroup is shown in **Table 4**. The characteristics of those individuals (n=2,104) being ascertained for the first time in IDEA through NDIA and born 1983-2015 are shown in **Table 5**.

**Table 3:** Ascertainment source for all cases with autism spectrum disorder, intellectual disability, or both in 25,487 individuals born between 1983 and 2015.

Ascertainment source	ASD with ID	ASD without ID	ID without ASD	Total
	n (row %*)			n (col%^)
DSC	107 (6.5)	403 (24.3)	1,146 (69.2)	1,656 (6.5)
DSC, Education	672 (18.1)	447 (12.0)	2,591 (69.8)	3,710 (14.6)
DSC, Education, NDIA	4,293 (59.5)	417 (5.8)	2,500 (34.7)	7,210 (28.3)
DSC, NDIA	379 (24.1)	644 (41.0)	547 (34.8)	1,570 (6.2)
Education	343 (6.3)	1,483 (27.3)	3,616 (66.4)	5,442 (21.4)
Education, NDIA	3,041 (80.1)	416 (11.0)	338 (8.9)	3,795 (14.9)
NDIA	56 (2.7)	1,631 (77.5)	417 (19.8)	2,104 (8.3)
Total	8,891 (34.9)	5,441 (21.3)	11,155 (43.8)	25,487

n, number of cases; DSC, Disability Services Commission; Education, Department of Education WA; NDIA, National Disability Insurance Agency; ASD, autism spectrum disorder; ID, intellectual disability.

*percentage of the row total; ^percentage of the column total.

**Table 4:** Characteristics of 5,899 individuals born between 1983 and 2015 who were identified through NDIA in 2020 but not previously registered with DSC.

Characteristic	ASD with ID	ASD without ID	ID without ASD	Total
	n (row %7)			n (col %^)
Age in 2020 years				
5-9	1,091 (64.3)	441 (26.0)	165 (9.7)	1,697 (28.8)
10-19	1,829 (54.3)	1,153 (34.2)	388 (11.5)	3,370 (57.1)
20-29	165 (24.3)	367 (54.1)	146 (21.5)	678 (11.5)
30-37	12 (7.8)	86 (55.8)	56 (36.4)	154 (2.6)
Total	3,097 (52.5)	2,047 (34.7)	755 (12.8)	5,899
Indigenous status				
Aboriginal	103 (31.4)	42 (12.8)	183 (55.8)	328 (5.6)
Non-Aboriginal	2,994 (53.7)	2,006 (36.0)	572 (10.3)	5,571 (94.4)
Total	3,097 (52.5)	2,047 (34.7)	55 (12.8)	5,899
Health Region				
Metro	2,485 (52.4)	1,706 (35.9)	554 (11.7)	4,744 (80.4)
Goldfields	46 (59.0)	16 (20.5)	16 (20.5)	78 (1.3)
Great Southern	65 (56.0)	41 (35.3)	10 (8.6)	116 (2)
Kimberley	20 (27.4)	18 (24.7)	35 (47.9)	73 (1.2)
Midwest	62 (49.6)	40 (32.0)	23 (18.4)	125 (2.1)
Pilbara	49 (60.5)	10 (12.3)	22 (27.2)	81 (1.4)
South West	292 (56.5)	162 (31.3)	63 (12.2)	517 (8.8)
Wheatbelt	77 (56.6)	35 (25.7)	24 (17.6)	136 (2.3)
Total	3,097^a^ (52.5)	2,047^b^ (34.7)	755^c^ (12.8)	5,899^d^

n, number of cases; NDIA, National Disability Insurance Agency; DSC, Disability Services Commission; ASD, autism spectrum disorder; ID, intellectual disability.

*percentage of the row total; ^percentage of the column total.

Number of cases with missing health region variable data: ^a^n=1; ^b^n=20; ^c^n=8; ^d^n=29.

**Table 5:** Characteristics of 2,104 individuals born between 1983 and 2015 who were not previously in IDEA but ascertained in 2020 only through NDIA.

Characteristic	ASD with ID	ASD without ID	ID without ASD	Total
	n (row %*)			n (col %^)
Age in 2020, years				
5-9	16 (3.4)	374 (79.1)	83 (17.5)	473 (22.5)
10-19	26 (2.3)	855 (76.8)	232 (20.8)	1,113 (52.9)
20-37	14 (2.7)	402 (77.6)	102 (19.7)	518 (24.6)
Total	56 (2.7)	1,631 (77.5)	417 (19.8)	2,104
Indigenous status				
Aboriginal	<5	37 (21.6)	130 (76)	171 (8.1)
Non-Aboriginal	52 (2.7)	1,594 (82.5)	287 (14.8)	1,933 (91.9)
Total	56 (2.7)	1,631 (77.5)	417 (19.8)	2,104

n, number of cases; IDEA, Intellectual Disability Exploring Answers database; NDIA, National Disability Insurance Agency; ASD, autism spectrum disorder; ID, intellectual disability.

*percentage of the row total; ^percentage of the column total.

**Figure 7:**
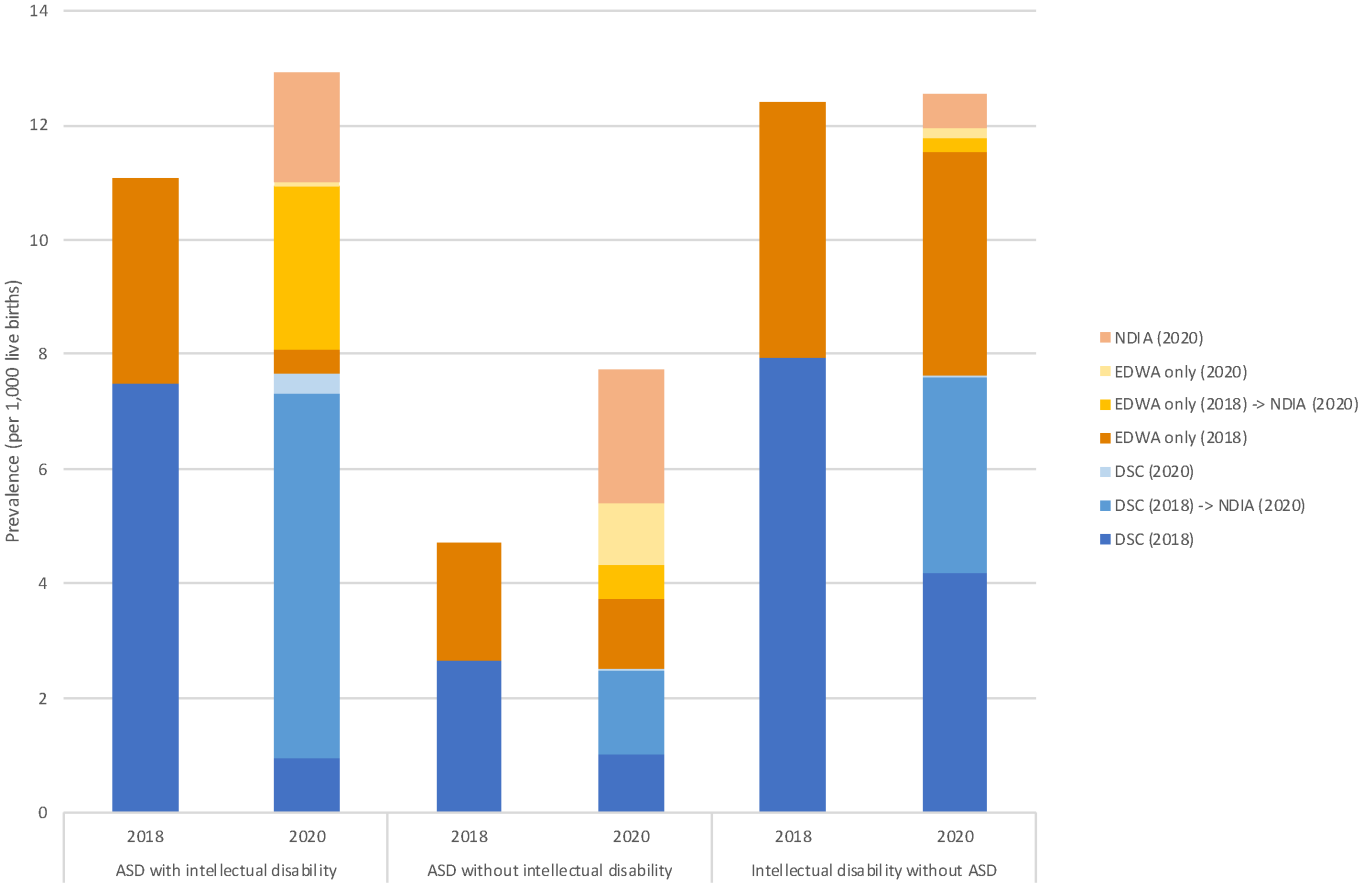
Prevalence of autism spectrum disorder with and without intellectual disability in Western Australia: a comparison between years 2018 and 2020. The figure provides a comparative analysis of the prevalence of autism spectrum disorder (ASD) with and without intellectual disability, alongside intellectual disability without ASD, per 1,000 live births in Western Australia for the ascertainment years 2018 and 2020. The data is further divided into different ascertainment sources: NDIA (National Disability Insurance Agency) for 2020, EDWA (Department of Education WA) for both 2018 and 2020, and DSC (Disability Services Commission) for 2018 and 2020. The arrow indicates the transition to NDIA access in 2020. The prevalence of intellectual disability without ASD slightly rose from 12.40 in 2018 to 12.54 in 2020, and for ASD with intellectual disability, from 11.08 to 12.92. Notably, the prevalence of ASD without intellectual disability nearly doubled, jumping from 4.71 to 7.75. Regarding ascertainment sources, in 2020, the primary source for case ascertainment was NDIA, accounting for the majority of newly identified or transitioned cases: 86.1% for ASD with intellectual disability and 57.0% for ASD without intellectual disability. In contrast, cases of intellectual disability without ASD had the lowest proportion of ascertainment from NDIA, at 34.0%. The denominator used to calculate prevalence differs by year and case group. For ASD with or without intellectual disability, live births from 1993-2013 (n=578,770) and 1993-2015 (n=648,247) were used for the calculations of prevalence in 2018 and 2020, respectively. In contrast, live births from 1983-2013 (n=821,460) and 1983-2015 (n=890,937) were used for intellectual disability without ASD.

The prevalence distribution, breakdown by ascertainment sources, of cases in IDEA in 2018 (prior to linkage to NDIA) and cases ascertained in 2020 (after linkage to NDIA data) is shown in **Figure 7**. Cases were grouped by diagnosis as ASD with intellectual disability, ASD without intellectual disability, or intellectual disability without ASD, to illustrate the variations in the observed changes. While the prevalence of intellectual disability without ASD saw a slight increase from 12.40/1,000 to 12.54/1,000, the change was more pronounced for the ASD with intellectual disability group, rising from 11.08/1,000 to 12.92/1,000. In contrast, the prevalence for the ASD without intellectual disability group nearly doubled from 4.71/1,000 to 7.75/1,000.

## Discussion

Our latest data for children born since 1983 shows that for intellectual disability, the prevalence has increased from 17/1,000 in 2010 to 22.5/1,000 in 2020 and for ASD (born since 1993) from 5.1/1000 to 20.67/1000. This is a fourfold increase for ASD and a modest increase for intellectual disability where a proportion of the children also have comorbid ASD. On the other hand, the prevalence of intellectual disability in males continues as before at twice that in females, as does the prevalence in Aboriginal people compared with non-Aboriginal people. The prevalence of ASD in males is three times that in females, whereas the prevalence in Aboriginal people is a third lower compared with non-Aboriginal people. The effects of colonization on Aboriginal culture and family connection have been cited as resulting in the high prevalence of disadvantage which has contributed to these increased rates of cognitive disability in the Aboriginal population (36).

One obvious question at least with respect to ASD is whether this is a true increase or whether this is affected by changes in diagnostic practices or source of ascertainment. We do know that in previous years in WA, an increase in the incidence of ASD diagnoses in children born between 1983 and 1999 did appear to relate to changes in diagnostic practices and services (13). However, our results show that the rise in ASD incidence is significantly steeper after 2012, despite international studies showing that the introduction of new criteria in DSM-5 in 2013, resulted in decreases or at most no change in the number diagnosed (37–41).

Over half of the 5,899 individuals who had not previously been receiving disability services are now receiving support through the NDIS on account of a diagnosis of ASD with co-existing intellectual disability, and a further one-third with a diagnosis of ASD without intellectual disability. About one-third of these children had been ascertained in the IDEA database previously through education sources only, but would not have been included in previous ASD estimates when ASD coding only from an educational source was not accepted in the IDEA database. Thus, we may have underestimated ASD prevalence at that time. It is also possible that others may have received a subsequent diagnosis of ASD, with the age of 71 months being the most frequently reported age of diagnosis under 7 years old in one Australian study (42). Similar to elsewhere (43), it may also be that some children have been diagnosed with language deficits, motor difficulties, or cognitive deficits prior to a later diagnosis of ASD.

It is important also to consider whether the observed trend is in line with global patterns. A recent systematic review demonstrated variability in prevalence by region, with an overall prevalence of 12/1,000 but as high as 20/1,000 in Australia, in keeping with our own findings (18). Recent surveillance studies of ASD in the United States (16) have indicated a much higher prevalence of 27.6/1,000 in children aged 8 years (with variation across states from 23.1/1,000 to 44.9/1,000) than the 8.3 per 1,000 reported for the Americas in this systematic review (18), possibly because the data were not only restricted to the USA but included Central and South America where access to diagnosis may be less.

Of note is that the Australian data contributing to the systematic review was derived from two studies using the Longitudinal Study of Australian Children (LSAC) as a sampling frame (44, 45). Notably, information regarding ASD diagnoses was provided by caregivers as opposed to clinicians and was not validated. Interestingly, the prevalence derived was much higher than in another Australian study using linked data from disability, hospital, and ambulatory mental health services in New South Wales, where a prevalence of 1.3% by 12 years was found (12). Nevertheless, the estimates from the LSAC study were similar to our own. Disability Services, through which the majority of ASD diagnoses were being made or validated in the past in WA, had a commitment to good clinical practice standards involving the contribution of multiple practitioners from different specialties (13). Our data show that there has been a sharp increase in the diagnosis of ASD, both with and without intellectual disability, since the addition of NDIA as a source of ascertainment. Taylor and others (11) in their survey of diagnostic practices in Australia showed that multidisciplinary teams had lower rates of diagnosis than sole clinicians. It is plausible that this reflects a higher degree of specificity available to multidisciplinary teams by way of the greater pooled expertise, and the appropriate withholding of a diagnostic label that is not an appropriate clinical descriptor. Multidisciplinary teams were seen in greater frequency in the public sector diagnostic services, and further, public sector diagnostic services had fewer diagnosticians ascribing an ASD diagnosis when individuals did not fulfill the criteria. And so we have to ask whether the diagnostic practices underpinning those cases ascertained in this study are markedly different from those employed historically. This paper which investigated the concordance of diagnosis between a “gold-standard” multidisciplinary team and a range of independent clinicians, with only about a quarter of participating clinicians receiving good levels of agreement with the original ASD assessment suggests perhaps not (46).

Also of note is the increased prevalence of ASD in Aboriginal children under 10 years although overall the prevalence is about two-thirds that of non-Aboriginal individuals. Whilst previous Australian studies have shown lower prevalence of ASD in Aboriginal children (47) an increase in ASD diagnosis for younger Aboriginal children may reflect a take-up of NDIS services (48). It may also indicate an increased awareness and confidence within the Aboriginal population of the possible benefits of earlier intervention for children with ASD. There was some variability of prevalence of both intellectual disability and ASD across Health Regions which may reflect an increased prevalence in areas of earlier introduction of the NDIS, where an NDIS trial site in the Perth Hills and a State Government version called NDIS My Way in the lower South West began in 2014 (49).

The NDIS is now the primary means of government funding to access disability supports in Australia. A recent study has suggested that the need for diagnoses to access disability supports via the NDIS could be contributing to increasing incidence rates of ASD within Australia, beyond the increases seen in other countries (20). Our results show clear increases over the past two decades, with an acceleration since the introduction of the NDIS. As we (13, 32) and others (50) have previously reported, changing diagnostic criteria, increased awareness of disability and diagnostic incentives to receive government funding have also been influencing factors. The relative contributions of these factors to the observed increases in prevalence is not clear. As previously discussed recent papers do not suggest that changes in the diagnostic criteria for ASD contributed to higher rates of diagnosis (40). The apparent increase in demand for individualized supports is likely a factor. However, families do not ascribe diagnostic outcomes for ASD to their children, ultimately this is a responsibility of clinicians and their diagnostic practices. The more modest rises in rates of intellectual disability in the IDEA database, when compared with ASD, disproportionately implicate diagnostic-specific contextual factors rather than or in addition to a broader demand for support. Thus, the concurrent relaxation of diagnostic practice standards in WA associated with the administration of access to the NDIS and the release of the National Guidelines appear to be important factors in understanding the rapid rise in prevalence. It remains unclear to what degree the additional cases ascertained by IDEA from the NDIS cohort represent identification of individuals who have ASD and require substantial government support in their daily lives, and what proportion may be receiving clinical labels for a range of disabilities with milder impacts to access government support that is not available elsewhere.

It seems implausible that such rapid increases in prevalence could reflect shifts in biological risk factors, with no known biological processes associated with ASD having undergone such substantial changes, time locked in some way, to the growth of ASD diagnosis in WA. However, we cannot ignore the fact that there could still be aetiological factors contributing to these increases. We recently undertook a systematic review to identify risk factors for intellectual disability (51). We grouped potential exposures into six topic areas: sociodemographic; antenatal and perinatal; maternal physical health; maternal mental health; environmental and genetic factors. Most of these topic areas also apply to ASD to a greater or lesser degree. It is important to consider whether changes in the prevalence of any relevant risk factors could be contributing to an increase in ASD prevalence. For example, we know that there is a temporal increase both in the prevalence of diabetes and mental health conditions and that there is an association between both maternal types 1 and 2 and gestational diabetes and maternal psychiatric disease and ASD in the offspring (52, 53).

Advancing paternal age has also been associated with an increase in ASD, with the offspring of men 50 years or older being twice as likely to have ASD compared with the offspring of men 29 years or younger (54), while there are various combinations of paternal and maternal age which specifically increase the risk (55). We also know that both have been increasing over time (35), so, particularly in relation to the father’s age, this could be making an aetiological contribution to the changing prevalence of ASD.

Finally, we need to consider the implications of this apparent epidemic in need for services especially for children vulnerable to developmental concerns and hence to the economy, especially in Australia but globally as well. The Australian system of disability support is unique - individualized funding for those facing significant economic costs relating to their disability is covered by the government through the NDIS (56). The wider community with disability should be able to access support in mainstream settings within the education, health and community sectors. Whilst the NDIS has resulted in improved outcomes and quality of life for many individuals with disability, supports for the wider community with disability may not have been sufficient to meet the increasing demand for services. It is likely this has resulted in a push for Australians to access individualized packages of support through the NDIS, giving rise to dramatically increasing costs of disability supports and sustainability concerns around the ongoing viability of the much-valued scheme.

The NDIS currently supports over 640,000 Australians and is projected to rise to over 1 million Australians by 2033. Almost two thirds of NDIS participants have a primary disability of ASD (35%), developmental delay (12%) or intellectual disability (15%). For children under the age of 18, the proportion is higher at 80% (51% for ASD, 21% for developmental delay and 8% for intellectual disability). The annual governmental spend on the NDIS is currently $35 billion and is projected to almost triple over the next decade. Participants with ASD account for a fifth and participants with intellectual disability account for a quarter of total scheme costs (57).

A recent governmental review of NDIS operations has recommended that supports for children with ASD and developmental concerns should be embedded into the educational infrastructure of government support rather than by means of individualized support packages (56). This is in line with the social model of disability where the needs of people with disability are integrated into mainstream systems of support. The review also suggests that this may result in future sustainability of an individualized funding scheme like the NDIS to support individuals, for whom the impact of disability is substantial and results in significant economic costs, to live a life of dignity with choice and control.

The role of diagnosis of neurodevelopmental disorders in informing eligibility for the NDIS also remains unclear. Recommendations have been made to focus on functional assessment rather than diagnosis to establish eligibility for and quantity of individualized funding. Implementation of this approach should consider risks of misdiagnoses and the potential influence of factors associated with the perceived need for services (58). The unintended incentivization of dysfunction that may occur is unlikely to align with the outcomes that all Australians hope to realize from the public investment in the NDIS. If the current mounting prevalence of ASD includes a substantial cohort of individuals for whom the diagnosis is ill fitting or inappropriate, the result may well be that the clinical utility of the ASD diagnostic label to drive good individual outcomes is progressively eroded. Efforts to promote appropriate diagnostic practices and minimize the ascription of inappropriate clinical diagnoses may prove a more pragmatic approach, retaining the best of the utility of diagnosis whilst managing the risks associated with it.

In light of these findings, the seemingly perpetual challenge for many countries, including Australia, who signed the UN Convention on the Rights of Persons with Disabilities (UNCRPD) in 2008, continues to be that of delivering high quality disability support that achieves outcomes for people with disability and their families in a sustainable way.

## Data availability statement

The datasets for this article are not publicly available due to concerns regarding participant/patient anonymity. Requests to access the datasets should be directed to the corresponding author.

## Ethics statement

The study was reviewed and approved by the Government of Western Australia Department of Health, Human Research Ethics Committee (Project 2014/24). Written informed consent from the participants was not required to participate in this study in accordance with the national legislation and the institutional requirements.

## Author contributions

JB: Conceptualization, Data curation, Formal analysis, Methodology, Validation, Writing – original draft, Writing – review & editing. RS: Investigation, Validation, Writing – review & editing. JJ: Investigation, Methodology, Writing – review & editing. MR: Investigation, Methodology, Writing – review & editing. KW: Data curation, Formal analysis, Methodology, Writing – original draft, Writing – review & editing. HL: Conceptualization, Investigation, Methodology, Writing – original draft, Writing – review & editing.
